# A systematic review of sham acupuncture validation studies

**DOI:** 10.1186/s12906-024-04506-1

**Published:** 2024-06-05

**Authors:** Sung Min Lim, Eunji Go

**Affiliations:** Department of Clinical Research on Rehabilitation, Korea National Rehabilitation Research Institute, 58 Samgaksan-Ro, Gangbuk-Gu, Seoul, 142-070 Republic of Korea

**Keywords:** Systematic review, Sham, Acupuncture, Validation

## Abstract

**Background:**

Acupuncture is widely used worldwide; however, studies on its effectiveness have been impeded by limitations regarding the design of appropriate control groups. In clinical research, noninvasive sham acupuncture techniques can only be applied through validation studies. Therefore, this systematic review aimed to evaluate the scope of existing literature on this topic to identify trends.

**Methods:**

We queried Pubmed, EMBASE, and the Cochrane Central Register of Controlled Trials databases from inception to July 2022 for relevant articles. Author names were used to identify additional relevant articles. Two independent reviewers assessed the identified articles based on the inclusion and exclusion criteria. The following data were extracted: study design, information regarding acupuncturists and participants, general and treatment-related characteristics of the intervention and control groups, participants’ experience of acupuncture, and research findings.

**Results:**

The database query yielded 673 articles, of which 29 articles were included in the final review. Among these, 18 involved the use of one of three devices: Streitberger (*n* = 5), Park (*n* = 7), and Takakura (*n* = 6) devices. The remaining 11 studies used other devices, including self-developed needles. All the included studies were randomized controlled trials. The methodological details of the included studies were heterogeneous with respect to outcomes assessed, blinding, and results.

**Conclusions:**

Sham acupuncture validation studies have been conducted using healthy volunteers, with a focus on blind review and technological developments in sham acupuncture devices. However, theren may be language bias in our findings since we could not query Chinese and Japanese databases due to language barriers. There is a need for more efforts toward establishing control groups suitable for various acupuncture therapy interventions. Moreover, there is a need for more rigorous sham acupuncture validation studies, which could lead to higher-quality clinical studies.

## Background

Acupuncture, a widely used therapy worldwide, involves inserting needles into the body for healing purposes [1]. Worldwide, numerous studies have been conducted to evaluate the efficacy of acupuncture; however, acupuncture-related clinical studies have been impeded by difficulties in designing an appropriate control group [2, 3]. When comparing the therapeutic efficacy of acupuncture and non-treatment controls, considering the general placebo effect and potential bias is crucial. Since the therapeutic efficacy of acupuncture is generally exaggerated, the specific effect of acupuncture remains to be established. To mitigate the problem regarding control groups, noninvasive sham acupuncture (SA) interventions, including the Streitberger's and Park sham needles, have been developed and used [4].

To facilitate the application of these noninvasive SA techniques in clinical research, relevant clinical validation studies are warranted. Accordingly, we aimed to conduct a systematic review of SA validation studies to investigate their characteristics, including participants, intervention and control group settings, and evaluation indicators. Our findings could inform the development and validation of novel and improved SA techniques.

## Methods

### Information sources and search engines

We performed a query of three databases (Pubmed, EMBASE, and the Cochrane Central Register of Controlled Trials) for relevant articles from inception to July 2022. We used the following search string: (acupuncture or needle) AND (sham or placebo) AND (validation or validity or validating or validate or credible or credibility). Author names were used to identify additional relevant articles. This study adheres to the PRISMA (Preferred Reporting Items for Systematic Reviews and Meta-Analyses) Statement, and the research protocol has been published in a previous paper [5].

### Selection criteria

To select eligible articles for this systematic review, two independent reviewers (SML and EJG) assessed the retrieved articles based on the following inclusion criteria: 1) original articles, 2) clinical trials, and 3) SA validation studies using SA control groups. We excluded studies unrelated to manual acupuncture or those testing the effects of acupuncture. In the primary title/abstract-based screening, articles considered irrelevant to the research topic were excluded. Subsequently, a secondary full-text screening was performed on articles with unclear abstracts. Disagreements were discussed until a consensus was reached.

### Data extraction and risk of *bias* assessment

Data extraction was conducted by two independent reviewers (SML and EJG) using a predetermined data extraction form. The following data were extracted from the selected studies: 1) study design; 2) information regarding acupuncturists and participants; 3) general and treatment-related characteristics of the intervention and control groups; 4) participants’ experience of acupuncture; and 5) research outcomes.

The literature quality was assessed using the Cochrane risk of bias assessment tool. The assessment items included random sequence generation (selection bias), allocation concealment (selection bias), blinding of participants and personnel (performance bias), blinding of outcome assessment (detection bias), incomplete outcome data (attrition bias), selective reporting (reporting bias), and other bias. Additionally, two researchers (SML and EJG) independently evaluated the literature quality, with disagreements resolved through discussion.

### Data analysis

Descriptive analyses (mean, standard deviation, and frequency analysis) were conducted on the outcomes of the SA validation studies.

## Results

### Search and article selection

**Fig.1:**
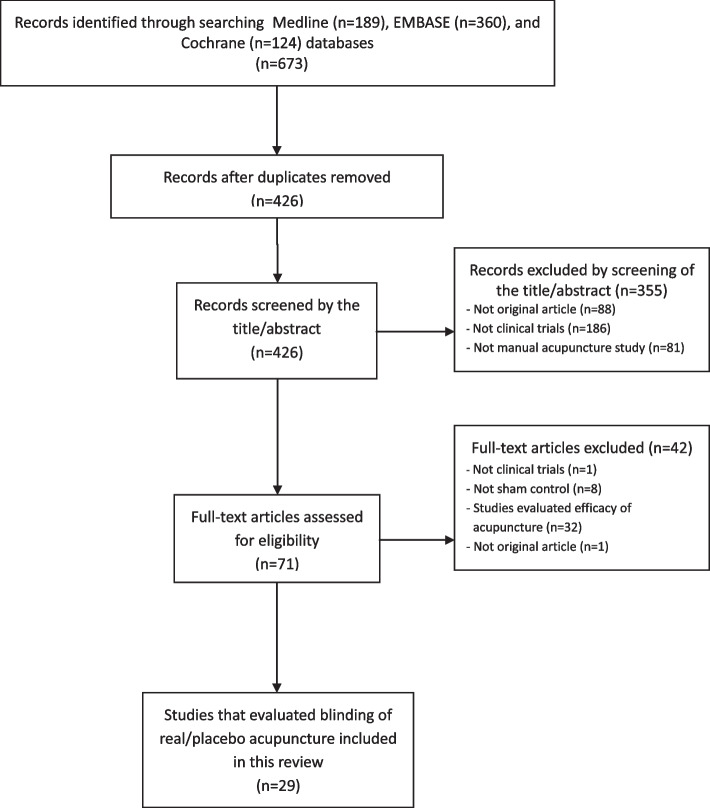
Flow chart of the trial selection process

The database query yielded 673 articles, of which 644 articles were excluded during the screening process based on title/abstract and full texts. Finally, 29 studies were included in this systematic review (Fig. 1) [6–34].

### Characteristics of the selected studies

The 29 selected articles were published between 1998 and 2016. Among them, five, seven, and six studies described validation tests for the Streitberger, Park, and Takakura devices, respectively. The remaining 11 studies described validation tests for other devices, including self-developed needles. Specifically, six studies used a blunted placebo needle and a block, cylinder, or pad foam [24, 26, 27, 30, 32, 33], one study used a toothpick and guide tube [25], two studies used an endermic acupuncture device with a flat, non-puncturing needle tip [28, 31], one study used a blunt, noninvasive needle that comprised a diamond honing stone and a guide tube [29], and one study used a sham device designed to prevent skin penetrations of needles using a hollow inner tube with a central base channel [34]. All studies were randomized controlled trials (RCTs) (Table 1).

**Table 1:** Summary of validation studies on sham acupuncture

Sham Device	Author (year)	Participants	Groups/Acupuncture points	Experience of acupuncture	Main Outcomes
Streitberger Device	Streitberger & Kleinhenz (1998) [6]	60 healthy volunteers	(a) AT, SA (*n* = 30), and LI4 (b) SA, AT (*n* = 30), and LI4	No	(1) Penetration (Yes/No, %) - AT: 90.0/10.0 (54/6) - SA: 78.3/21.7 (47/13) (2) Pain (mean ± SD) - AT: 13.40 ± 10.58 - SA: 8.86 ± 10.55 (3) Deqi (Yes/No, %) - AT: 56.7/43.3 (34/26) - SA: 21.7/78.3 (13/47)
	White PL et al (2003) [7]	37 patients with chronic pain	(a) AT, SA (*n* = 18), and pain located on the hip and knee (b) SA, AT (*n* = 19), and pain located on the hip and knee	No	(1) Deqi - No significant difference between acupuncture types (p > 0.05) during needle sensation using a VAS score (dull, radiating, stinging, and electric) (2) Penetration (Yes/No/DK, %) - AT: 67.6/27.0/5.4 (25/10/2) - SA: 59.5/47.8/2.7 (22/14/1)
	White P et al (2007) [8]	20 healthy volunteers	(a) AT, SA (*n* = 10), and LI4 (b) SA, AT (*n* = 10), and LI4	Mixed	(1) Blinding (correct/incorrect, %) - AT: 80.0/20.0 (16/4) - SA: 50.0/50.0 (10/10) (2) Penetration (Yes/No, %) - AT: 95.0/5.0 (19/1) - SA: 75.0/25.0 (15/5) (3) NSQ (Mean ± SD) - AT: 5.30 ± 5.97 - SA: 2.65 ± 3.03
		14 patients with chronic pain	(c) AT, SA (*n* = 7), and LI4 (d) SA, AT (*n* = 7), and LI4		(1) Blinding (correct/incorrect, %) - AT: 100.0/0.0 (14/0) - SA: 78.6/21.4 (11/3) (2) Penetration (Yes/No, %) - AT: 78.6/21.4 (11/3) - SA: 64.4/35.6 (9/5) (3) NSQ (Mean ± SD) - AT: 12.29 ± 7.44 - SA: 5.14 ± 6.70
	Enblom A et al (2008) [9]	80 healthy volunteers	(a) AT (*n* = 40), PC6 (b) SA (*n* = 40), PC6	No	(1) Blinding (correct/incorrect/DK, %) - AT: 32.5/12.5/55 (13/5/22) - SA: 32.5/22.5/45 (13/9/18) (2) Pain (Severe/Moderate/mild/No) - AT: 0.0/6.7/50.0/43.3 (0/2/15/13) - SA: 3.3/3.3/36.7/56.7 (1/1/11/17)
	Xie CC et al (2013) [10]	60 healthy volunteers	(a) AT, SA (*n* = 30), BL23 (b) SA, AT (*n* = 30), BL23	Yes	(1) Penetration (Yes/No/DK, %) - AT: 75.0/25.0/0.0 (45/15/0) - SA: 81.7/18.3/0.0 (49/11/0) (2) Pain - No significant difference between acupuncture types (p > 0.05) (3) Deqi (Yes/No/DK, %) - AT: 40.0/60.0/0.0(24/36/0) - SA: 23.3/76.7/0.0(14/46/0)
Park Device	Park J et al (2002) [11]	58 patients with stroke	(a) AT (*n* = 29), LI4 (b) SA (*n* = 29), LI4	No	(1) Blinding (correct/incorrect/DK, %) - AT: 37.9/0.0/62.1 (11/0/18) - SA: .0.0/31.0/69.0 (0/9/20)
		40 healthy volunteers	(c) AT (*n* = 21), LI4 (d) SA (*n* = 19), LI4		(1) Deqi (Yes/No, %) - AT: 80.9/18.1 (17/4) - SA: 5.3/94.7 (1/18)
		60 healthy volunteers	(e) AT (*n* = 31), LI4 (f) SA (*n* = 29), LI4		(1) Deqi (Yes/No, %) - AT: 71.0/29.0 (22/9) - SA: 20.9/79.1 (6/23)
	Tsukayama et al (2006) [12]	20 healthy volunteers	(a) AT, SA (*n* = 14), and LI4 (b) SA, AT (*n* = 6), and LI4	Yes	(1) Penetration (Yes/No/DK, %) - AT: 100.0/0.0/0.0 (20/0/0) - SA: 35.0/60.0/5.0 (7/12/1) (2) Deqi (Yes/No/DK, %) - AT: 70.0/25.0/5.0 (14/5/1) - SA: 20.0/75.0/5.0 (4/15/1)
			(c) AT, SA (*n* = 10), and BL23 (d) SA, AT (*n* = 10), and BL23		(1) Penetration (Yes/No/DK, %) - AT: 70.0/30.0/0.0 (14/6/0) - SA: 50.0/40.0/10.0 (10/8/2) (2) Deqi (Yes/No/DK, %) - AT: 40.0/55.0/5.0 (8/11/1) - SA: 10.0/80.0/10/0 (2/16/2)
	Tan CWC et al (2009) [13]	20 healthy volunteers	(a) AT, SA (*n* = 20), PC3, PC4, PC5, and PC6	No	(1) Blinding (correct/incorrect, %) - AT: 75.0/25.0 - SA: 62.0/38.0
			(b) AT, SA (*n* = 20), and non-traditional points		(1) Blinding (correct/incorrect, %) - AT: 28.0/72.0 - SA: 74.0/26.0
	Chae Y et al (2011) [14]	14 healthy volunteers	(a) AT, SA (*n* = 14), and LI4	Yes	(1) Power of insertion (mean ± SD, gf) - AT: 68.5 ± 12.2 - SA: 27.2 ± 3.9 (2) Blinding (correct/incorrect, %) - AT: 78.6/21.4 (11/3) - SA: 85.7/14.3 (12/2) (3) Penetration (mean ± SD) - AT: 4.9 ± 3.1 - SA: 1.7 ± 2.3 (4) Pain (mean ± SD) - AT: 4.9 ± 2.7 - SA: 1.9 ± 2.6 (5) Deqi (mean ± SD) - AT: 3.6 ± 2.7 - SA: 1.5 ± 2.7
	Lee H et al (2011) [15]	79 healthy volunteers	(a) AT (*n* = 39), LI4 (b) SA (*n* = 40), LI4	Mixed	(1) Blinding (correct/incorrect/DK, %) - AT: 64.1/23.1/12.8(25/9/5) - SA: 45.0/52.5/2.5(18/21/1) (2) Penetration (mean ± SD) - AT: 3.8 ± 2.8 - SA: 2.0 ± 1.7 (3) Deqi - Significant difference in hurting and soreness levels between acupuncture types (*p* < 0.05) during needle sensation assessment on a 10-point numeric rating scale
			(c) AT (*n* = 39), CV12 (d) SA (*n* = 40), CV12		(1) Blinding (correct/incorrect/DK, %) - AT: 23.1/53.8/23.1 (9/21/9) - SA: 42.5/42.5/15.0 (17/17/6) (2) Penetration (mean ± SD) - AT: 2.4 ± 2.1 - SA: 2.4 ± 2.2 (3) Deqi - No significant difference between acupuncture types (p > 0.05) during needle sensation assessment on a 10-point numeric rating scale
			(e) AT (*n* = 39), ST36 (f) SA (*n* = 38), ST36		(1) Blinding (correct/incorrect/DK, %) - AT: 41.0/33.3/25.7 (16/13/10) - SA: 57.9/31.6/10.5 (22/12/4) (2) Penetration (mean ± SD) - AT: 2.7 ± 2.4 - SA: 2.2 ± 2.0 (3)) Deqi - No significant difference between acupuncture types (p > 0.05) during needle sensation assessment on a 10-point numeric rating scale
	Liang ZH (2013) [16]	60 healthy volunteers	(a) AT, SA (*n* = 30), and BL23 (b) SA, AT (*n* = 30), and BL23	Yes	(1) Pain - In group A, significant differences were observed between acupuncture types (*p* < 0.05) (2) Penetration (Yes/No, %) - AT: 73.3/26.7 (44/16) - SA: 80.0/20.0 (48/12) (3) Deqi (Yes/No, %) - AT: 58.3/41.7 (35/25) - SA: 38.3/61.7 (23/37)
	To & Alexander (2016) [17]	16 healthy volunteers	(a) AT, SA (*n* = 16), LI4, LI10, LI11, LI14, LI15, and TE14	Mixed	(1) Blinding (correct/incorrect, %) - AT: 41.9/58.1 (18/29) - SA: 54.7/45.3 (25/24)
		14 patients with SIS	(b) AT (*n* = 8), SI3, SI12, LV3, GB21, and ST38 (c) SA (*n* = 6), SI3, SI12, LV3, GB21, and ST38		(1) Blinding - All participants thought they were receiving AT
Takakura Device	Takakura & Yajima (2007) [18]	10 acupuncturists	(a) AT, SA (*n* = 40), and LI4	Yes	(1) Blinding (correct/incorrect/DK, %) - AT: 37.1/42.3/20.6 (63/72/35) - SA: 46.5/40.9/12.6 (107/94/29)
		60 healthy volunteers	(b) AT, SA (*n* = 60), and TE5		(1) Penetration (Yes/No, %) - AT: 80.0/20.0 (48/12) - SA: 41.7/58.3 (25/35) (2) Deqi (Yes/No, %) - AT: 80.0/20.0 (48/12) - SA: 33.3/66.7 (20/40)
	Takakura & Yajima (2008) [19]	One acupuncturist	(a) AT, SA (*n* = 114), and TE5	Yes	(1) Blinding (correct/incorrect/DK, %) - AT: 37.7/59.6/2.7 (43/68/3) - SA: 47.4/47.4/5.2 (54/54/6)
		114 healthy volunteers			(1) Blinding (correct/incorrect, %) - AT: 68.4/31.6 (78/36) - SA: 43.9/56.1 (50/64) (2) Penetration (Yes/No, %) - AT: 63.2/36.8 (72/42) - SA: 63.2/36.8 (72/42) (3) Penetration (median (mean)) - AT: 1.9 (1) - SA: 2.1 (1) (4) Deqi (Yes/No, %) - AT: 35.1/64.9 (40/74) - SA: 26.3/73.7 (30/84) (5) Deqi (median (mean)) - AT: 1.3 (0) - SA: 1.0 (0)
	Takakura et al (2010) [20]	10 acupuncturists	(a) AT, SA1 (skin touch), SA2 (non-touch) (*n* = 100), and LI4	Yes	(1) Blinding (correct/incorrect/DK, %) - AT: 47.0/39.0/14.0 (47/39/14) - SA1: 23.0/64.0/13.0 (23/64/13) - SA2: 34.0/49.0/17.0 (34/49/17) (2) ‘Feeling of needle insertion’ was the most important criterion for judgment
	Takakura et al (2011) [21]	One acupuncturist	(a) AT, SA1 (skin touch), SA2 (non-touch) (*n* = 80), and three points on forearm	Yes	(1) Blinding (correct/incorrect/DK, %) - AT: 62.5/37.5/0.0 (50/30/0) - SA1: 41.3/57.5/1.2 (33/46/1) - SA2: 46.3/53.7/0.0 (37/43/0)
		80 healthy volunteers			(1) Blinding (correct/incorrect/DK, %) - AT: 81.3/16.2/2.5 (65/13/2) - SA1: 47.5/46.3/6.2 (38/37/5) - SA2: 71.3/22.5/6.2 (57/18/5) (2) Penetration (Yes/ST/No/DK, %) - AT: 71.3/10.0/0.0/0.0 (57/8/0/0) - SA1: 33.8/13.7/0.0/2.5 (27/11/0/2) - SA2: 0.0/2.5/0.0/0.0 (0/2/0/0) (3) Deqi (Yes/No, %) - AT: 41.3/58.7 (33/47) - SA1: 16.3/83.7 (13/67) - SA2: 2.5/97.5 (2/78)
	Takakura N et al (2013) [22]	One acupuncturist	(a) AT, SA (*n* = 109), TE5	Yes	(1) Blinding (correct/incorrect, %) - AT: 53.2/46.8 (58/51) - SA: 44.0/56.0 (48/61)
		109 healthy volunteers			(1) Blinding (correct/incorrect, %) - AT: 78.0/22.0 (85/24) - SA: 59.6/40.4 (65/44) (2) Pain (Yes/No, %) - AT: 59.6/40.4 (65/44) (3) Pleasure (Yes/No, %) - AT: 16.5/83.5 (18/91) (4) Unpleasure (Yes/No, %) - AT: 33.0/67.0 (36/73)
	Takakura N et al (2013) [23]	One acupuncturist	(a) AT, SA1 (skin touch), SA2 (non-touch) (*n* = 80)	Yes	(1) Blinding (correct/incorrect/DK, %) - AT: 35.0/60.0/5.0 (28/48/4) - SA1: 27.5/67.5/5.0 (22/54/4) - SA2: 28.8/68.7/2.5 (23/55/2)
		80 healthy volunteers			(1) Blinding (correct/incorrect/DK, %) - AT: 60.0/36.2/3.8 (48/29/3) - SA1: 53.8/38.7/7.5 (43/31/6) - SA2: 71.2/17.5/11.3 (57/14/9) (2) Pain (Yes/No, %) - AT: 60.0/40.0 (48/32) - SA1: 50.0/50.0 (40/40) (3) Deqi (Yes/No, %) - AT: 45.0/55.0 (36/44) - SA1: 17.5/82.5 (14/66) - SA2: 3.8/96.2 (3/77)
Other Device	Fink MG et al. (2001) [24]	68 patients with headache	(a) AT (*n* = 34), GB20, LI4, LR3, and TW5 (b) SA (*n* = 34), GB20, LI4, LR3, and TW5	No	(1) Psychological checklist - No significant difference between acupuncture types (p > 0.05) (2) Penetration (Yes/No, %) - AT: 100.0/0.0 (32/0) - SA: 87.5/12.5 (28/4) (3) Deqi (Yes/No, %) - AT: 84.4/15.6 (27/5) - SA: 34.4/65.6 (11/21)
	Sherman KJH et al (2002) [25]	10 healthy volunteers	(a) AT, SA (*n* = 10), UB23, DU3, DU4, SI3, UB40, and KI3	No	(1) Blinding - No significant difference between acupuncture types (p > 0.05)
		52 patients with CBP	(b) AT (*n* = 23), DU3, UB23, UB40, and KI3 (c) SA (*n* = 29), DU3, UB23, UB40, and KI3		(1) Blinding (a 5-point scale, %) - AT: 30/35/26/9/0 - SA: 0/21/28/38/14 (2) Penetration (Yes/No/DK, %) - AT: 83.0/17.0/0.0 - SA: 69.0/17.0/14.0 (3) Remove (Yes/No/DK, %) - AT: 43.3/43.3/13.4 - SA: 41.3/48.7/10.0
	Fink MK (2005) [26]	10 healthy volunteers	(a) AT, SA (*n* = 10), and LI4	Mixed	(1) Pain (Yes/No, %) - AT: 100.0/0.0 (20/0) - SA: 100.0/0.0 (20/0) (2) Deqi (Yes/No, %) - AT: 85.0/15.0 (17/3) - SA: 60.0/40.0 (12/8) (3) Spread of deqi (Yes/No, %) - AT: 45.0/55.0 (9/11) - SA: 45.0/55.0 (9/11)
	Goddard GS et al (2005) [27]	40 healthy volunteers	(a) AT (*n* = 20), LI4 (b) SA (*n* = 20), LI4	No	(1) Blinding (correct/incorrect, %) - AT: 80.0/20.0 (16/4) - SA: 60.0/40.0 (12/8)
	Kim S (2008) [28]	20 acupuncturists 60 healthy volunteers	(a) AT or SA (*n* = 80), LI4 (check acupuncture before treatment)	Yes	(1) Blinding (correct/incorrect, %) * Appearance - Acupuncturists: 25.0/75.0 - Participants: 45.0/55.0 * Receiving treatment - Acupuncturists: 45.0/55.0 - Participants: 50.0/50.0
			(b) AT or SA (*n* = 80), LI4 (check acupuncture after treatment)		(1) Blinding (correct/incorrect, %) * Receiving treatment - Acupuncturists: 65.0/35.0 - Participants: 63.3/36.7 * Appearance - Acupuncturists: 65.5/34.5 - Participants: 48.3/51.7
			(c) AT or SA (*n* = 30), LI4 (can check acupuncture) (d) AT or SA (*n* = 30), LI4 (cannot check acupuncture)		(1) Blinding (correct/incorrect, %) - Intervention group: 66.7/33.3 - Control group: 60.0/40.0
	Tough EAW et al (2009) [29]	37 patients with RWI	(a) AT (*n* = 19) (b) SA (*n* = 18)	Mixed	(1) Blinding (correct/incorrect/DK, %) - AT: 52.6/0.0/47.4 (10/0/9) - SA: 5.5/72.2/22.3 (1/13/4) (2) Psychological checklist - No significant difference between acupuncture types (p > 0.05) (3) Sensation of acupuncture - Aching and heavy sensations were reported at a higher proportion in true acupuncture (15/3)
	Kreiner MZ et al (2010) [30]	32 healthy volunteers	(a) AT, SA (*n* = 16), and LI4 (b) SA, AT (*n* = 16), and LI4	No	(1) Penetration (Yes/No, %) - AT: 65.6/34.4 (21/11) - SA: 28.1/71.9 (9/23) (2) Blinding (correct/incorrect, %) - AT: 84.4/15.6 (27/5) - SA: 25.0/75.0 (8/24) (3) Deqi (Yes/No, %) - AT: 43.7/56.3 (14/18) - SA: 34.4/65.6 (11/21) (4) Pain (Yes/No, %) - AT: 9.4/90.6 (3/29) - SA: 0.0/100.0 (0/32) (5) Comfortable (Yes/No, %) - AT: 93.7/6.3 (30/2) - SA: 100.0/0.0 (32/0)
			(c) AT, SA (*n* = 16), and ST6 (d) SA, AT (*n* = 16), and ST6		(1) Penetration (Yes/No, %) - AT: 68.8/31.2 (22/10) - SA: 28.1/71.9 (9/23) (2) Blinding (correct/incorrect, %) - AT: 84.4/15.6 (27/5) - SA: 18.7/81.3 (6/26) (3) Deqi (Yes/No, %) - AT: 46.9/53.1 (15/17) - SA: 34.4/65.6 (11/21) (4) Pain (Yes/No, %) - AT: 6.3/93.7 (2/30) - SA: 6.3/93.7 (2/30) (5) Comfortable (Yes/No, %) - AT: 100.0/0.0 (32/0) - SA: 100.0/0.0 (32/0)
	Lee SL et al (2012) [31]	Seven smokers	(a) AT + SA (*n* = 7), HT8, and KL2	Mixed	(1) Facial temperature - Significant difference between acupuncture types (p < 0.05) (2) Blinding - Three out of seven participants responded correctly (42.9%)
	Liu BX et al (2014) [32]	60 healthy volunteers	(a) AT, SA (*n* = 30), LI4, BL36, RN12, and BL25 (b) SA, AT (*n* = 30), LI4, BL36, RN12, and BL25	Mixed	(1) Penetration - No significant difference between acupuncture types (p > 0.05) (2) Sensation of acupuncture - Significant difference in distension between acupuncture types (p < 0.05) (3) Pain - Significant difference between acupuncture types (p < 0.05) (4) Acceptance - The placebo needle was more easily accepted (odd ratio = 1.63,1.01–2.64)
	Wong ELL et al (2015) [33]	18 healthy volunteers	(a) AT, SA1 (sham point) (*n* = 18), LI4, ST36	Mixed	(1) Blinding (LI4) (correct/incorrect, %) - AT: 88.9/11.1 (16/2) - SA: 83.3/16.7 (15/3) (2) Blinding (ST36) (correct/incorrect, %) - AT: 88.9/11.1 (16/2) - SA: 50.0/50.0 (9/9)
			(b) AT, SA2 (minimal penetration) (*n* = 18), LI4, and ST36		(1) Blinding (LI4) (correct/incorrect, %) - AT: 83.3/16.7 (15/3) - SA: 55.6/44.4 (10/8) (2) Blinding (ST36) (correct/incorrect, %) - AT: 83.3/16.7 (15/3) - SA: 50.0/50.0 (9/9)
			(c) AT, SA3 (special device) (*n* = 18), LI4, and ST36		(1) Blinding (LI4) (correct/incorrect, %) - AT: 77.8/22.2 (14/4) - SA: 16.7/83.3 (3/15) (2) Blinding (ST36) (correct/incorrect, %) - AT: 72.2/27.8 (13/5) - SA: 11.1/88.9 (2/16)
	Juel JL et al (2016) [34]	14 healthy volunteers	(a) AT, SA (*n* = 14), RM4, RM 5, RM 7, RM9, RM10, and RM 12	Mixed	(1) Blinding - 5 out of 14 participants responded correctly (35.7%) (2) Visceral pain stimulation - No significant difference between acupuncture types (p > 0.05) (3) Electroencephalography (EEG) - No significant difference between acupuncture types (p > 0.05)

*RCT* Randomized control trial, *SIS* Shoulder impingement syndrome, *CBP* Chronic back pain, *RWI* Recent whiplash injury, *AT* Acupuncture therapy, *SA* Sham acupuncture, *ST* Skin touch, *NSQ* Needling sensation questionnaire, *DK* Don’t know, *Remove* Removal of the needle or sham needle, *Facial temperature* Measurement of facial temperature using DITI (digital infrared thermal imaging), *Acceptance* acceptability of needle using a 5-point scale, *Visceral pain stimulation* pain scores and rectal balloon volumes following rectal balloon distensions

Regarding participants, 21 studies involved healthy adults, with seven studies (including all studies that used the Takakura device) attempting to blind the acupuncturists. Among the remaining eight studies, four involved patients and four involved both healthy adults and patients. Moreover, 17 studies included both intervention and control groups, while 12 administered both acupuncture therapy (AT) and SA to the intervention group. Notably, three studies that used the Takakura device performed validation experiments on two SA types: skin-touch and non-touch.

The most frequently used acupoint for SA validation was LI4, followed by BL23, TE5, and ST36. Further, 14 and 13 studies involved single and multiple acupoints, respectively. Four of the 13 studies that used multiple acupoints assessed acupoint-dependent differences in outcomes. Two studies did not mention the acupoint chosen.

Acupuncture manipulation was performed in 21 studies. Four studies used a Streitberger device [7–10], five studies used a Park device [11, 12, 15–17], six studies used a Takakura device [18–24], and six studies used other devices [25, 26, 29, 30, 32, 34]. The manipulation method was usually rotation.

Twenty studies considered the participants’ acupuncture experience. Among them, 11 and nine studies recruited participants with and without acupuncture experience, respectively. The most frequently used SA validation method was guessing the applied acupuncture type (*n* = 21). Other SA validation methods included penetration, pain, and deqi sensation.

### Reliability of acupuncturist blinding

All six studies that used the Takakura device evaluated acupuncturist blinding, with one study using a different device. These studies tested whether the acupuncturists could correctly guess the AT type after administering two (AT/SA) or three (AT/skin-touch SA/non-touch SA) different acupuncture treatments by providing a guessed (correct/incorrect) or “don’t know” (DK) response.

Among the studies that used the Takakura device, incorrect and DK answers outnumbered correct answers in four [18–20, 23] and two studies [21, 22] with AT and SA treatments, respectively, suggesting that the Takakura device is effective in acupuncturist blinding. In studies that identified three AT types, non-touch SA led to more incorrect answers than skin-touch SA [20, 21, 23]. In the study that used a different device, the rate of incorrect and correct answers was higher when the needle was shown before and after treatment, respectively [28].

In the study conducted by Takakura et al. [20], participants were instructed to indicate the reason for the answer, with the most frequent reason being deqi sensation.

### Reliability of participant blinding

Participant blinding was evaluated in two, five, four, and eight studies using the Streitberger, Park, Takakura, and other devices, respectively. In all these studies, the participants were instructed to answer in the same aforementioned format as the acupuncturists. Among these studies, the rate of incorrect answers was higher for AT and SA in four [14, 15, 17, 28] and 14 [8, 11, 13, 15, 19, 21–23, 25, 27, 29, 30, 33, 34] studies, respectively. In the remaining study, most participants gave the answer ‘DK’, which contributed to a low rate of correct answers for SA [9].

Two studies compared the blinding success according to the selected acupoint. Participants were more likely to correctly guess the acupuncture type when it was administered to the upper limbs (vs. lower limbs), limbs (vs. torso), and traditional acupoints (vs. non-traditional acupoints) [13, 30]. Chae et al. [14] measured the penetrating force using a computerized system and observed that it was associated with the blinding outcome.

### Blinding Index

The blinding effect was analyzed in 24 studies, with five studies being excluded owing to failure to provide data for calculating the Blinding Index [35] (Table 2). Among these, 11 studies had blinding scenarios of “unblinded” and “opposite guess” in the experimental (AT) and control (SA) arms, respectively. Additionally, two studies had a blinding scenario of “random guess” in both arms. Accordingly, 13 of the 24 (54%) studies were considered to have applied effective blinding scenarios. Moreover, six studies were unblinded in the experimental arm (AT), and random guessing was applied in the control arm (SA), while three studies were unblinded in both arms. Furthermore, one study applied random guessing in the experimental arm (AT) and was unblinded in the control arm (SA), while another study applied random and opposite guessing in the experimental (AT) and control (SA) arms, respectively (Table 3).

**Table 2:** Blinding index values computed from 24 validation studies

Sham Device	Author (year)	N	VBI	VBI 95% CI	SBI	SBI 95% CI	Scenario
Streitberger Device	Streitberger & Kleinhenz (1998) [6]	60	0.8	0.65 to 0.95	-0.57	-0.78 to -0.36	Unblinded/opposite
	White PL et al. (2003) [7]	37	0.41	0.12 to 0.69	-0.22	-0.53 to 0.09	Unblinded/opposite
	White P et al. (2007) [8]	34	0.76	0.55 to 0.98	0.24	-0.09 to 0.56	Unblinded/unblinded
	Enblom A et al. (2008) [9]	80	0.20	0.00 to 0.40	0.10	-0.13 to 0.33	Random/random
	Xie CC et al. (2013) [10]	60	0.50	0.28 to 0.72	-0.63	-0.83 to -0.44	Unblinded/opposite
Park Device	Park J et al. (2002) [11]	58	0.38	0.20 to 0.56	-0.31	-0.48 to -0.14	Unblinded/opposite
	Tsukayama et al. (2006) [12]	20	0.70	0.48 to 0.92	0.07	-0.22 to 0.37	Unblinded/random
	Tan CWC et al. (2009) [13]	20	0.03	-0.11 to 0.17	0.36	0.23 to 0.49	Random/unblinded
	Chae Y et al. (2011) [14]	14	0.57	0.14 to 1.00	0.71	0.35 to 1.08	Unblinded/unblinded
	Lee H et al. (2011) [15]	79	0.06	-0.10 to 0.22	0.06	-0.11 to 0.23	Random/random
	Liang ZH (2013) [16]	60	0.47	0.24 to 0.69	-0.60	-0.80 to -0.40	Unblinded/opposite
	To M & Alexander (2016) [17]	30	0.05	-0.20 to 0.30	-0.24	-0.48 to 0.00	Random/opposite
Takakura Device	Takakura & Yajima (2007) [18]	60	0.60	0.40 to 0.80	0.17	-0.08 to 0.42	Unblinded/random
	Takakura & Yajima (2008) [19]	114	0.37	0.20 to 0.54	-0.12	-0.30 to 0.06	Unblinded/random
	Takakura N et al. (2011) [21]	80	0.8	0.65 to 0.95	-0.57	-.0.78 to -0.36	Unblinded/opposite
	Takakura N et al. (2013) [22]	109	0.41	0.12 to 0.69	-0.22	-0.53 to 0.09	Unblinded/opposite
	Takakura N et al. (2013) [23]	80	0.76	0.55 to 0.98	0.24	-0.09 to 0.56	Unblinded/unblinded
Other Device	Fink MG et al. (2001) [26]	64	1.00	1.00 to 1.00	-0.75	-0.98 to -0.52	Unblinded/opposite
	Sherman KJH et al. (2002) [25]	52	0.65	0.34 to 0.96	-0.52	-0.80 to -0.24	Unblinded/opposite
	Goddard GS et al. (2005) [27]	40	0.60	0.25 to 0.95	0.20	-0.23 to 0.63	Unblinded/unblinded
	Tough EAW et al. (2009) [29]	37	0.53	0.30 to 0.75	-0.67	-0.93 to -0.40	Unblinded/opposite
	Kreiner MZ et al. (2010) [30]	32	0.69	0.51 to 0.87	-0.56	-0.77 to -0.36	Unblinded/opposite
	Liu BX et al. (2014) [32]	60	0.93	0.88 to 0.97	-0.87	-0.93 to -0.80	Unblinded/opposite
	Wong ELL et al. (2015) [33]	18	0.56	0.17 to 0.94	-0.67	-1.01 to -0.32	Unblinded/opposite

*VBI* blinding index of real acupuncture group, *SBI* blinding index of sham acupuncture group

**Table 3:** Blinding scenarios

Experimental arm	Control arm	Possible interpretations (on blinding and treatment effectiveness)	Trials number (%)
Random guess	Random guess	Ideal	2 (8.3)
Random guess	Opposite guess	(Psychologically/behaviorally) Rare	1 (4.2)
Random guess	Unblinded	Relatively rare – possible, little treatment effect, and completely no effect in control arms (for example, no placebo effect)	1 (4.2)
Unblinded	Unblinded	Could be problematic. Possible, clear treatment effect in the experimental arm and no treatment effect in the control arm (for example, patients know what to expect)	3 (12.5)
Unblinded	Opposite guess	Ideal (for example, patients tend to have wishful thinking or patients do not know how to control treatment looks)	11 (45.8)
Unblinded	Random guess	Could be problematic. Possible, clear treatment effect in the experimental arm and no treatment effect in the control arm (for example, patients do not know what to expect in the absence of treatment)	6 (25)
Opposite guess	Opposite guess	Rare or unlikely	0
Opposite guess	Random guess	Rare	0
Opposite guess	Unblinded	Possible, no treatment effect or patients tend to be negative or unmotivated	0

### Participants’ responses to acupuncture-related sensations

Twenty studies evaluated participants’ acupuncture-related sensations. Among these, five, four, five, and six studies used the Streitberger, Park, Takakura, and other devices, respectively. Participants were asked to rate the acupuncture-related sensations, including pain and penetration, on a 1–10 or 1–100 visual analog scale (VAS).

Fifteen studies evaluated the participants’ penetration sensation. Among these, 12 and three studies evaluated the presence/absence and level of penetration sensation, respectively. Eleven studies performed pain evaluation, of which four and seven studies evaluated the presence/absence and level of pain, respectively. In six studies that reported the penetration sensation, most participants perceived the penetration in both AT and SA. The perception of penetration sensation was lesser in SA and AT in six [6–8, 24, 25, 32] and two [10, 16] studies, respectively. In four studies, more participants reported the penetration sensation only with AT [12, 18, 21, 30]. Notably, in the studies conducted by Chae et al. [14] and Lee et al. [15], participants who received AT and SA in the LI4 acupoint reported significantly stronger penetration sensation with AT; however, no significant differences were observed in the CV12 and ST36 acupoints [15, 19].

Takakura et al. [22] reported that most participants experienced pain with both AT and SA; however, the perceived pain was lesser in SA. Fink [26] showed that all participants reported pain with both AT and SA. In contrast, Kreiner et al. [30] reported that only 7.8% and 3.1% of the participants felt pain with AT and SA, respectively. Another study showed that 59.6% of the participants reported only AT-induced pain [22]. Regarding the pain level, three studies reported stronger pain in AT than in SA [6, 9, 14]. Liang et al. [16] reported that only group A (AT → wash out → SA) perceived significantly stronger pain with AT. In the remaining three studies, the pain level did not significantly differ between AT and SA [10, 32, 34]. Moreover, responses were sought regarding the feelings of relief, pleasure, facial temperature, acceptability, and comfort. Notably, only the facial temperature measurements showed differences between AT and SA.

### Participants’ report on deqi sensation

Fifteen studies evaluated the participants’ deqi sensation, of which three, five, four, and three studies used the Streitberger, Park, Takakura, and other devices, respectively. Notably, twelve and three studies evaluated the presence/absence and level of deqi sensation, respectively. Six studies reported greater deqi sensation with AT than with SA [6, 11, 12, 16, 18, 24]. Five studies reported that most patients lacked deqi sensations with AT, which was even lower with SA [10, 19, 21, 23, 30]. Fink et al. [26] showed that 84.4% and 34.4% of participants reported deqi sensation with AT and SA, respectively. Chae et al. [14] reported that participants felt significantly stronger deqi sensations with AT than with SA. White et al. [7] reported no differences between the two groups. Lee et al. [15] reported some differences in deqi sensation at LI4 but no differences between the two groups at CV12 or ST36.

### Quality assessment

**Fig. 2:**
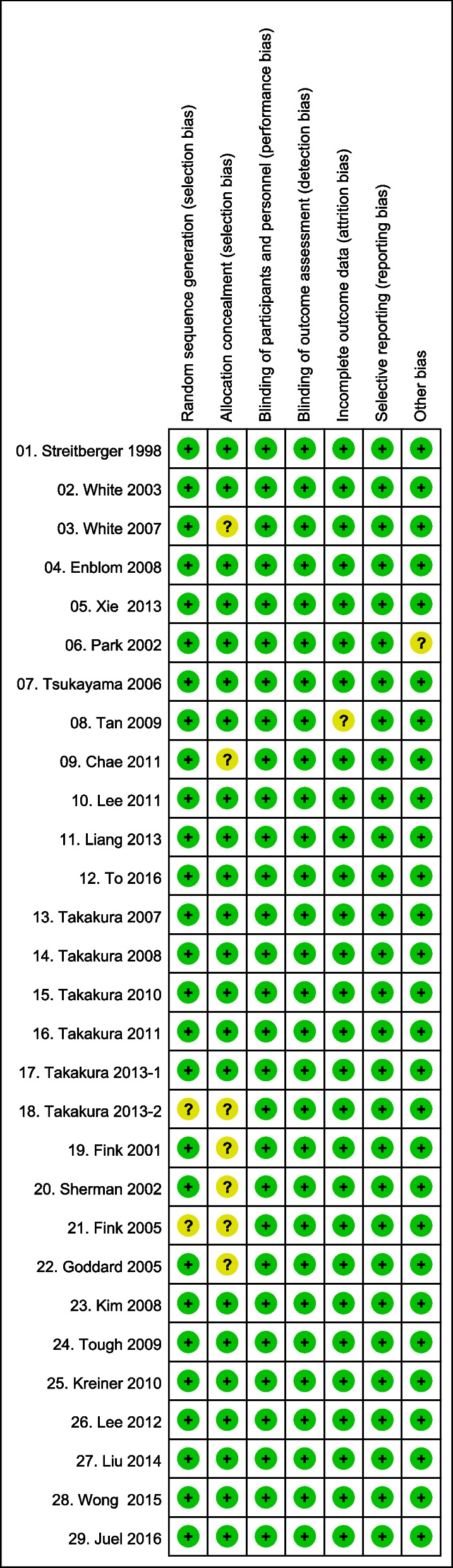
Risk of bias summary

Figure 2 presents the results of the assessment items of the overall risk of bias. In all included studies, 192 “low risk” and 11 “unclear risk” assessments were performed in seven domains. The risk of bias was low for random sequence generation (selection bias), allocation concealment (selection bias), blinding of participants and personnel (performance bias), blinding of outcome assessment (detection bias), incomplete outcome data (attrition bias), selective reporting (reporting bias), and other bias in 27, 22, 29, 29, 28, 29, and 28 studies, respectively. Among the assessment items, allocation concealment (selection bias) had the highest frequency of “unclear risk” evaluation (n = 7) due to the lack of a specific description of the method of concealing the allocation sequence. Random sequence generation (selection bias) had the second highest frequency of “unclear risk” evaluation (n = 2) due to an unmentioned or unclear randomization method. Similar distributions were noted for the low and unclear risks of bias in studies using the Streitberger, Park, and Takakura devices.

## Discussion

Invasive control groups involving needle insertion into an area other than a traditional acupuncture point or a traditional acupuncture point unrelated to the treatment objective may be unsuitable as placebo control groups since the procedure can induce physiological effects similar to invasive AT [36]. Noninvasive SA needles were developed to overcome these limitations. Noninvasive SA devices, including the Streitberger, Park, and Takakura devices, are characterized by blunt needle tips that cannot penetrate the skin but have the same shape as needles used for AT, which ensures participant blinding [4]. Validation studies on SA devices used across acupoints and participants are important for improving acupuncture-related clinical research that involves SA control groups [37, 38].

All included SA validation studies in this review had an RCT design involving randomly assigned intervention (AT) and control (SA) groups of healthy volunteers or patients. Blinding was influenced by the participants’ acupuncture experience, acupuncturist’s experience, acupoint, and type of SA (skin-touch or non-touch). A higher rate of blinding success was observed for participants without acupuncture experience, experienced acupuncturists, acupoints in body parts other than the hand, non-traditional acupoints, and skin-touch SA. Including DK as a response option may influence the results and their interpretation; therefore, this should be carefully considered.

Other aspects of blinding that were evaluated included penetration, pain, and deqi sensations. Specifically, the presence/absence and level of sensations were evaluated through yes/no responses and a VAS, respectively. Although the evaluation items for deqi varied across studies, it was mostly evaluated based on the level of sensations such as dull pain, heat, stinging, and tingling. Since AT- and SA-related sensations are important factors in studies involving patients, future studies should comprehensively consider the influence of the disease on sensations based on validation study outcomes using healthy volunteers.

In clinical studies evaluating the therapeutic effect of AT, establishing an appropriate control that allows the exclusion of the placebo effect is important, and thus, evaluation of the AT-specific effects. However, in real practice, precise assessment of the AT-specific effects is difficult owing to the multiple and complex factors that influence the AT-related experiences and expectations of patients [39]. Therefore, using an SA control intervention that allows effective blinding of patients and assessment of AT-specific effects is crucial for obtaining highly reliable clinical findings [40]. Meta-analyses conducted by Vickers et al. [41, 42] revealed that the AT intervention group showed clinically significant outcomes compared with the SA control group, which indicates that appropriate SA controls can allow high-quality clinical evidence. Moreover, compared with noninvasive SA interventions, penetration of a real acupuncture needle can achieve a significant analgesic effect for a specific condition such as pain [43]. Therefore, future SA-controlled clinical trials that use the optimal AT protocol and adequate sample size for the desired effect size could further improve evidence-based medicine. Additionally, for RCTs that include a no-intervention group, it would be helpful for validation of the SA control.

According to White et al. [8], compared to healthy participants, patients experience a stronger needle sensation for both real and sham needles and are more likely to report both as real needles. Thus, differences in sensation during AT or differences in treatment expectations between patients and healthy participants could affect the results. Consequently, generalizing the results of validation studies for sham needles in healthy adults or patients could be inappropriate. Future studies should focus on identifying the most suitable sham needles for specific diseases.

SA devices that involve skin contact or minimal insertion may pose limitations in controlled clinical studies owing to potential neurophysiological effects via skin contact or SA. Ideally, SA controls should have physical features and psychological effects identical to those of AT, which minimizes the physiological effects on the human body and maintains blinding of both participants and acupuncturists even in long-term clinical studies. Since SA validation studies are conducted using a single- or double-randomized design, establishing suitable control groups, including electroacupuncture and intradermal acupuncture, for various AT interventions is crucial to validate their therapeutic efficacy.

A limitation of this study is the possibility of language bias since we did not query Chinese and Japanese databases due to language barriers.

## Conclusions

More efforts are required to establish control groups suitable for various acupuncture therapy interventions. Moreover, more rigorous sham acupuncture validation studies are necessary, potentially improving the quality of clinical studies.

## Data Availability

All data generated or analyzed during this study are included in this published article.

## Abbreviations

AT: Acupuncture therapy
CBP: Chronic back pain
DK: Don’t know
NSQ: Needling Sensation Questionnaire
RCT: Randomized controlled trial
RWI: Recent whiplash injury
SA: Sham acupuncture
SIS: Shoulder impingement syndrome
ST: Skin touch
VAS: Visual analogue scale
